# Microbial taxa and functional genes shift in degraded soil with bacterial wilt

**DOI:** 10.1038/srep39911

**Published:** 2017-01-04

**Authors:** Hongchun Zhang, Rui Wang, Shu Chen, Gaofu Qi, Zhili He, Xiuyun Zhao

**Affiliations:** 1 College of Life Science and Technology, Huazhong Agricultural University, Wuhan 430070, China; 2 Institute for Environmental Genomics and Department of Microbiology and Plant Biology, University of Oklahoma, Norman, OK 73019, USA

## Abstract

Soil degradation is a serious global problem, but little is known about how soil microbial communities respond to soil degradation as well as their feedback to ecosystem functioning. In this study, we found the microbial community composition, structure and functional potential significantly altered in the degraded soils with bacterial wilt (termed as degraded soils). Compared with healthy soils, OTU richness of beneficial microorganisms were significantly decreased, but OTU richness of pathogenic microorganisms were significantly increased in the degraded soils. Functional gene array (GeoChip 5.0) analysis showed the functional metabolic potential of genes involved in stress, virulence, sulfur cycle, metal resistance, degradation of plant cell wall was significantly increased in the degraded soils. Increased functional metabolic potential of these genes may be related to the acidification and severe plant disease of degraded soils. Biological activity of degraded soils was obviously decreased with weakened soil enzyme activities when compared to the healthy soils. Soil pH and enzyme activities were negatively correlated with the abundance of genes involved in sulfur cycle, virulence, and stress responses. This study provides new insights into our understanding of soil microbial community responses to soil degradation.

Soil degradation is a serious global environmental problem. Long-term continuous cropping and excessive use of chemical fertilizers have caused a serious decline in soil quality^1^. About 40% of the world agricultural soil is seriously degraded whereas 24% area of the productive soil is still under continuous degradation^2^. The lost cost for land degradation is estimated to be US$40 billion per year. Soil degradation is the decline of soil quality, including physical, chemical and biological deterioration such as loss of organic matter, decline in soil fertility and structural conditions, erosion, soil acidification and salinization, nutrient imbalance, soil compaction, and loss of soil biodiversity^3^,^4^. In China, the long-term highly-intensive land use and agricultural chemical overuse have led to the degradation of soil quality, which are reflected by the unbalanced soil nutrients, soil acidification, pollutant accumulation, and biodiversity deterioration^5^,^6^,^7^. Severe soil degradation also results in an accumulation of soil-borne pathogens, which directly affect the growth and yield of crops. For example, the percentage of peanut stems infected by *Sclerotium rolfsii* was higher at soil pH 5.6 than in alkaline soil. Poor soil aeration caused by poor soil structure was associated with the development of carrot cavity spot disease caused by *Pythium* spp.^8^,^9^. Based on the data of international application system analysis and research institute, the total area of degraded soils in China is about 4.65 × 10^9^ hm^2^, presenting a great threat to human survival and sustainable development^6^.

The study of soil quality has been largely focused on soil fertility, soil physical and chemical properties. Microorganisms play an important role in the cycling of soil nutrients and decomposition of soil minerals, and they are also important indicators of soil quality because of their important roles in maintaining soil fertility and rapid responses to environmental changes^10^. Metagenomic analysis has provided new insights into our understanding of soil microbial community responses to global changes (*e.g.*, warming, elevated CO_2_, altered precipitation) and various environmental fluctuations in the natural or man-made ecosystems^11^,^12^,^13^,^14^. Application of high throughput sequencing-based and microarray-based metagenomic technologies can rapidly obtain useful informations related to microbial community structure and functional potential. For example, the field exposure of a grassland ecosystem to elevated CO_2_ dramatically altered the structure and functional potential of soil microbial communities^12^, and the elevated CO_2_ and O_3_ significantly affect the functional composition, structure and metabolic potential of soil microbial communities^13^. 16S rRNA gene pyrosequencing and a functional gene array of GeoChip 4.0, was used to investigate the shifts of microbial composition and functional gene structure in the paddy soils with different fertilization treatments, and found the balanced chemical fertilization was beneficial for soil microbial community and its functions^14^. Zhao *et al*. also analyzed the changes of soil microbial community simulated by soil transplant and maize cropping via GeoChip 3.0^15^.

Long-term continuous monoculture of crops such as corn, sorghum, soybean, wheat, and tobacco, can result in severe soil degradation in China^16^,^17^. Tobacco grows rapidly with excessive request for soil nutrients. After plantation with tobacco, the fields will become barren with outbreak of severe soil-borne diseases such as tobacco bacterial wilt. Here, we selected the field with continuous monoculture of tobacco for >10 years as a model to investigate the mechanism of soil degradation. The soil-borne diseases such as bacterial wilt caused by *Ralstonia solanacearum*, was very popular in these degraded soils. In this study, the soils with high disease incidence of tobacco bacterial wilt were selected and characterized as degraded soils, and the soils without tobacco bacterial wilt were selected as healthy soils.

In this study, we hypothesized that: (1) the taxonomic and functional composition and structure of soil microbial communities would be shifted in the degraded soils of tobacco field, and (2) the functional metabolic potential of key genes related to stress responses, metal resistance, sulfur (S) cycle, virulence, and degradation of plant cell wall would be increased in the degraded soils of tobacco field due to poor soil environments. To test these hypotheses, we analyzed soil microorganisms by functional gene array (GeoChip 5.0) and MiSeq sequencing of 16S rRNA and 18S rRNA gene amplicons, and then compared the microbial community composition and structure between healthy and degraded soils of tobacco field. Our results showed the functional metabolic potential of genes involved in virulence, stress responses, S cycle, and degradation of plant cell wall were generally enhanced in the degraded soils than the healthy soils. The results provide new insights into our understanding of soil microbial community responses to soil degradation and possible feedback mechanisms to ecosystem functioning.

## Results

### Soil biological, chemical and physical properties

The biological, chemical and physical properties of soils are listed in Table 1. The content of soil available P of degraded soils was significantly (*P* < 0.05) lower than the healthy soils. The activities of urease (*P* < 0.01), sucrase (*P* < 0.01) and catalase (*P* < 0.05) of degraded soils were all significantly lower than the healthy soils, indicating the biological activities in degraded soils were much lower than the healthy soils. The pH of healthy soils was significantly (*P* < 0.05) higher than the degraded soils. Compared to the degraded soils, the urease activity and pH value increased by 38.5% and 0.63 units in the healthy soils, respectively. However, there was no obvious change in available N, K, organic matter between the degraded and healthy soils. Soil moisture and soil porosity were higher, and soil bulk density was lower in the healthy soils than the degraded soils, but without significant difference between them.

### Incidence of bacterial wilt disease and plant growth

*R. solanacearum* causes serious bacterial wilt in tobacco. In the degraded soils, 85.48% of tobacco plants were infected by *R. solanacearum*; however, no tobacco plant was infected by *R. solanacearum* in the healthy soils. Also, the disease index of bacterial wilt was significantly (*P* < 0.01) higher in the degraded soils than the healthy soils. Tobacco seedlings grew better, and the plant height and stem girth were significantly (*P* < 0.05) higher in the healthy soils than the degraded soils (Table 2).

### Microbial diversity and community structure

Based on 16S rRNA gene sequences, the bacterial community richness and diversity was analyzed. The bacterial richness (Chao1) and diversity (Shannon indexes) were all higher in healthy soils than in degraded soils. Otherwise, the healthy soils were found with more OTUs than the degraded soils (Table S1).

**Figure 1:**
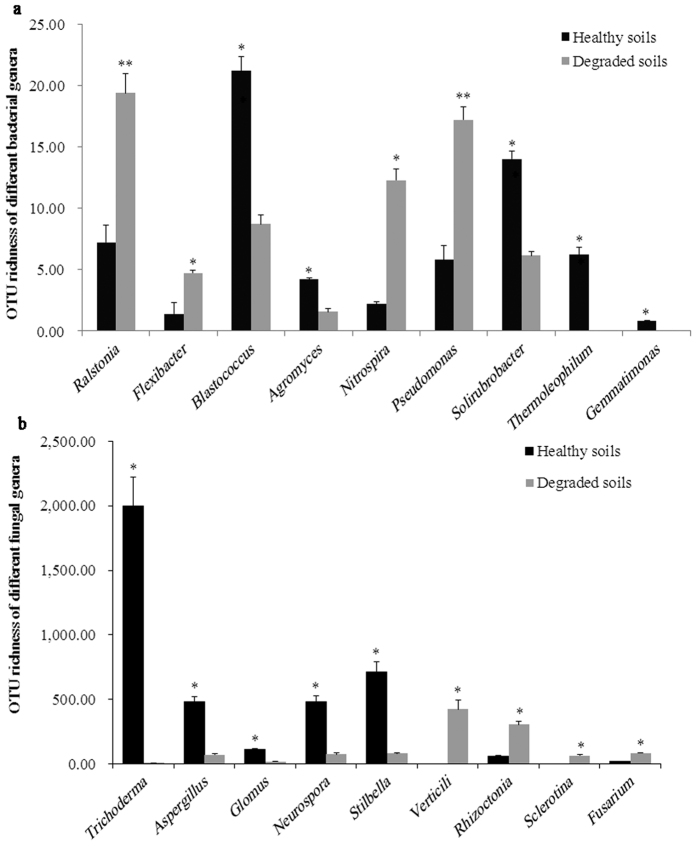
Analysis of microbial abundance in healthy and degraded soils. (**a**) Comparison of abundance of different bacteria between healthy and degraded soils. (**b**) Comparison of abundance of different fungi between healthy and degraded soils. All data are presented as the mean ± SE. Bars with asterisk (*) indicate significant (*P* < 0.05) difference between healthy and degraded soils.

*Proteobacteria* was the most dominant bacterial phylum observed accounting for 34.2% of the sequences, and *Acidobacteria* was the second most abundant phylum (33.3%). Thereby, these two phyla accounted for about 67.5% of the sequences in the soil. In the degraded soils, a higher relative abundance was found for *Proteobacteria, Acidobacteria*, and *Bacteroidetes* when compared to the healthy soils, indicating the microbial community diversity in the degraded soils was different from the healthy soils at the phylum level. In the healthy soils, the relative abundance of *Actinobacteria* and *Firmicutes* was significantly (*P* < 0.05) higher than the degraded soils (Fig. S1). At the genus level, the OTU richness of beneficial *Blastococcus, Agromyces, Solirubrobacter, Thermoleophilum* and *Gemmatimonas* were significantly (*P* < 0.05) increased in the healthy soils when compared to the degraded soils (Fig. 1a). In the healthy soils, a significantly (*P* < 0.05) lower relative abundance was found from pathogens of *Ralstonia* (*e.g. R. solanacearum*) and *Pseudomonas* (*e.g. P. corrugate*, P*. mediterranea, P, aeruginosa* and *P. syringae*), nitrifying bacteria of *Nitrospira* and denitrifying bacteria of *Flexibacter* when compared to the degraded soils (Fig. 1a, Table S3).

The fungal richness (Chao1) and diversity (Shannon indexes) were both higher in healthy soils than in degraded soils, and the healthy soils were found with more fungal OTUs than the degraded soils (Table S2). *Ascomycota* was the most dominant fungal phylum observed, accounting for 61.8% of the sequences. *Basidiomycota* was the second most abundant phylum, accounting for 21.5% of the sequences (Fig. S2). In the healthy soils, the relative abundances of beneficial *Trichoderma, Aspergillus, Glomus, Neurospora* and *Stilbella* were significantly (*P* < 0.05) higher than the degraded soils (Fig. 1b), while the relative abundances of pathogenic *Verticili, Rhizoctonia, Sclerotina*, and *Fusarium* were significantly (*P* < 0.05) lower than the degraded soils (Fig. 1b).

### GeoChip analysis of key functional genes

**Figure 2:**
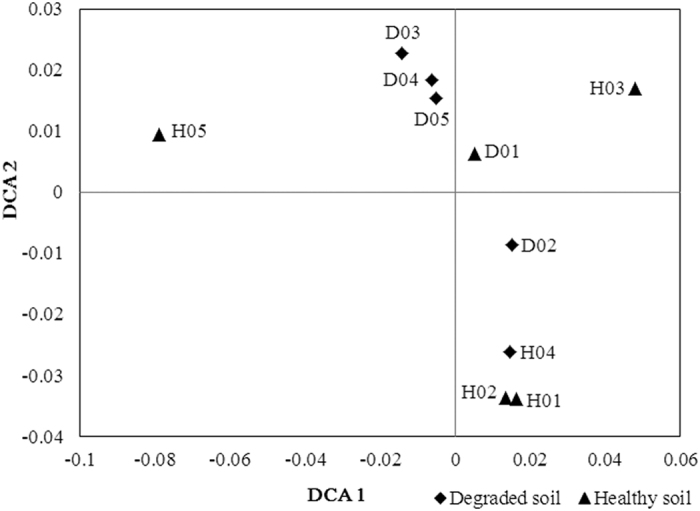
Detrended Correspondence Analysis (DCA) of microbial communities of healthy and degraded soils based on functional genes detected.

To determine whether the overall microbial community functional structure, DCA was performed based on all detected functional genes. As shown in Fig. 2, the four degraded soil samples (D01, D03, D04, and D05) were clustered together and well separated from the healthy soils. D02 was separated from the healthy soil samples at DCA2. The three healthy soils (H01 H02, and H04) were clustered together and separated from the degraded soils samples. Other two healthy soils (H03 and H05) were separated from the degraded soil samples at DCA1.

In the degraded soils, the functional metabolic potential of genes involved in carbon cycling (carbon degradation, carbon fixation, methane), nitrogen cycling (ammonification, nitrification, denitrification, nitrogen fixation, N assimilation, assimilatory N reduction, dissimilatory N reduction), phosphorus cycling (polyphosphate degradation, phytic acid hydrolysis, polyphosphate synthesis) were similar to the healthy soils (Fig. S3), indicating these genes involved in C, N, P cycles are not the main factors to cause soil degradation.

#### Stress response genes

**Figure 3:**
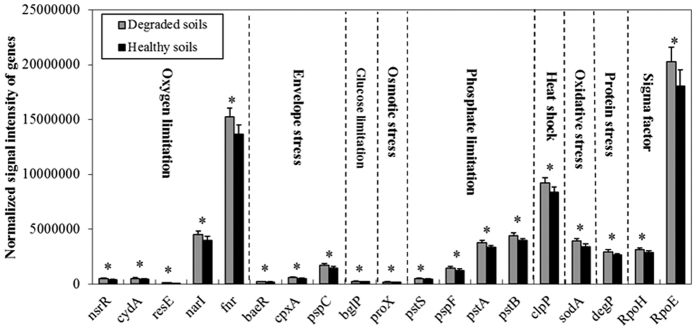
Comparison of functional metabolic potential of stress related genes between healthy and degraded soils. All data are presented as the mean ± SE. Bars with asterisk (*) indicate significant (*P* < 0.05) between healthy and degraded soils.

Most of stress response genes, including envelope stress, oxygen limitation, phosphate limitation, protein stress and sigma factors, had higher functional metabolic potential in the degraded soils than the healthy soils (Fig. S4). Especially, the functional metabolic potential of genes involved in oxygen limitation (*nsrR, cydA, resE, narI, fnr*), envelope stress (*baeR, cpxA, pspC*), glucose limitation (*bglP*), osmotic stress (*proX*), phosphate limitation (*pstS, pspF, pstA, pstB*), heat shock (*clpP*), oxidative stress (*sodA*), protein stress (*degP*), and sigma factors (*rpoH, rpoE*) were significantly (*P* < 0.05) enhanced in the degraded soils than those in the healthy soils (Fig. 3), indicating the environment is very poor for microorganisms in the degraded soils. Oxygen limitation means soil hardening deterioration, glucose or phosphate limitation indicates poor available soil nutrients, and osmotic stress suggests unbalanced ion in the degraded soils. As a result, the microorganisms are impacted by different stresses such as oxidative, osmotic, envelope and protein stress, accompanying with enhanced stress response genes in the degraded soils.

#### Virulence genes

**Figure 4:**
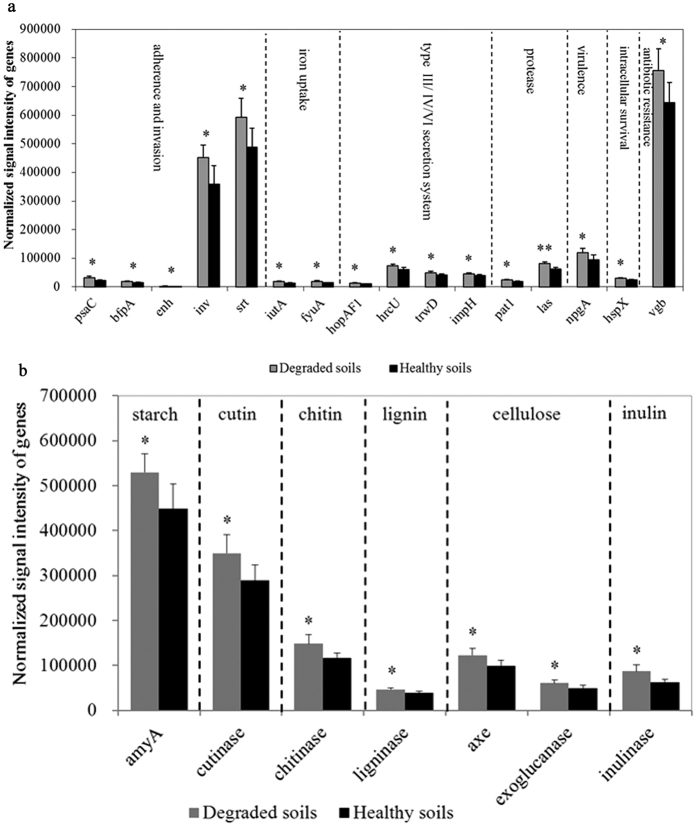
Comparison of functional metabolic potential of virulence related genes and degradation of plant cell wall genes between healthy and degraded soils. (**a**) Virulence related genes. (**b**) Genes for degradation of plant cell wall. All data are presented as the mean ± SE. Bars with asterisk (*) and double asterisks (**) indicate significant (*P* < 0.05) and very significant (*P* < 0.01) difference between healthy and degraded soils, respectively.

Most of virulence genes were more abundant in the degraded soils than in the healthy soils, including toxin, type II, III, IV, VI and VII secretion system, and virulence proteins. The functional metabolic potential of genes involved in toxin, protease and type VI secretion system were significantly (*P* < 0.05) enhanced in the degraded soils when compared to the healthy soils (Fig. S5). The functional metabolic potential of genes involved in adherence, invasion and colonization (*psaC, bfpA, enh, inv, srt*), iron uptake (*iutA, fyuA*), type III secretion system (*hopAF1, hrcU*), type IV secretion system (*trwD*), type VI secretion system (*impH*), protease (*pat1, las*), virulence (*npgA*), intracellular survival (*hspX*), and antibiotic resistance (*vgb*) were significantly (*P* < 0.05) enhanced in the degraded soils than in the healthy soils (Fig. 4a). These virulent genes were necessary for pathogens to infect and survive in plants, and the higher functional metabolic potential of these virulence genes means the more abundant plant pathogens in the degraded soils.

#### Degradation of plant cell wall genes

Most of plant cell wall degradation genes were more abundant in the degraded soils, including those derived from pathogens such as *Ralstonia, Pseudomonas, Erwinia, Xanthomonas, Verticillium, Sclerotinia* and *Fusarium*. The functional metabolic potential of genes involved in degradation of starch (*amyA*), cutin (cutinase), chitin (chitinase), lignin (ligninase), cellulose (*axe*, exoglucanase), inulin (inulinase) were significantly (*P* < 0.05) enhanced in the degraded soils when compared to the healthy soils (Fig. 4b). Cell wall is involved in the innate immunity of plants, which is very important for protection from pathogens infection. Thereby, the enhanced genes for degradation of plant cell wall are beneficial for pathogens to infect tobacco in the degraded soils, verified by an outbreak of plant diseases in the field (Table 2).

#### Sulfur cycling genes

**Figure 5:**
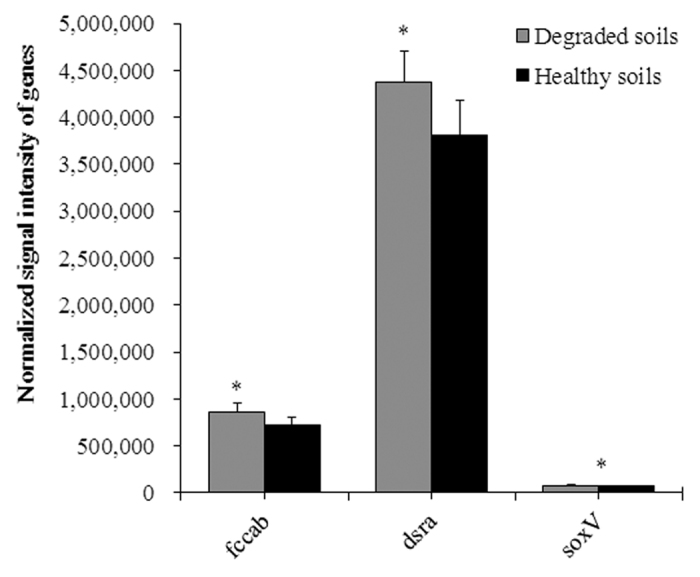
Comparison of functional metabolic potential of sulfur cycling related genes between healthy and degraded soils. All data are presented as the mean ± SE. Bars with asterisk (*) indicate significant (*P* < 0.05) difference between healthy and degraded soils.

Some sulfur cycling genes were more abundant in the degraded soils than the healthy soils. The functional metabolic potential of genes involved in sulfide oxidation (*fccab*), sulfur oxidation (*soxV*) and sulfite reduction (*dsra*) were significantly (*P* < 0.05) enhanced in the degraded soils when compared to the healthy soils (Fig. 5). Generally, the enhanced sulfur cycling genes are found in the acidic soils, so the enhanced sulfur cycling genes mean the acidification of degraded soils (Table 1).

#### Metal resistance genes

Most of metal resistance genes were more abundant in the degraded soils than the healthy soils. The functional metabolic potential of genes involved in transport and detoxification of arsenic (*aoxb, arsB*), chromium (*chrA, chrr*), copper (*copA*), sodium (*natB, nhaA, nhaB, nhaD*), zinc (*msc2, zntA*), nickel (*nikA, nreB*), magnesium (*mgtA, mgtE*), and iron (*cirA, fecA, feoB*) were significantly (*P* < 0.05) enhanced in the degraded soils when compared to the healthy soils (Fig. S6). This is also closely related to high content of heavy metals in the degraded soils (data unpublished here).

### Relationships of soil microbial community structure with soil environmental and functional properties

**Figure 6:**
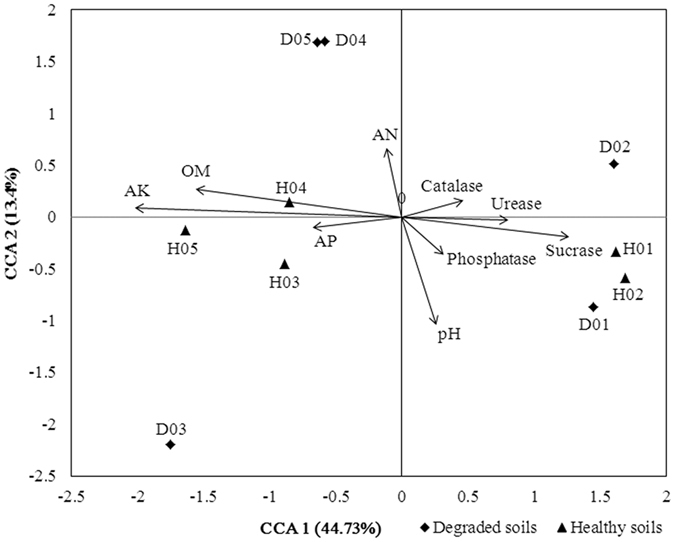
Canonical correspondence analysis (CCA) of relationship between microbial community functional structure and soil properties. The environmental variables are indicated with arrows, including pH, urease, phosphatase, sucrase, catalase, organic matter (OM), available potassium (AK), available nitrogen (AN), and available phosphorous (AP). H01-05: healthy soils; D01-05: degraded soils.

The relationships between microbial community functional structure and soil properties were assessed by canonical correspondence analysis (CCA) (Fig. 6). Nine parameters including phosphatase activity, urease activity, sucrase activity, catalase activity, pH, available phosphorous (AP), available nitrogen (AN), available potassium (AK) and organic matter (OM), were selected for analysis by CCA. Over 58% of the community functional variation could be explained by the above-selected variables. The first canonical axis was positively correlated with urease activity, sucrase activity and catalase activity, and negatively correlated with AK, OM and AP. The second axis was positively correlated with AN, and negatively correlated with soil pH and phosphatase activity. Since the longer arrows represent the more important variables, the variables of AK, OM, soil pH and sucrase activity appeared to play the major roles in shaping soil microbial functional structure. In addition, the microbial functional structures in H03, H04 and H05 were positively correlated with AK, OM and AP, while H01 and H02 were positively affected by activities of urease, phosphatase and sucrase.

### Correlations between functional gene abundances and soil environmental factors

Mantel tests were performed between functional structure and soil variables. Soil urease activity was significantly (*P* < 0.05) negatively correlated with the genes involved in S cycle, including *cysJ, cysI, dsra, fccab* and *soxV* (Table 3). Many genes involved in stress responses were significantly (*P* < 0.05) negatively correlated with soil pH, urease activity, sucrase activity and catalase activity. Soil pH, urease activity, sucrase activity and catalase activity were significantly (*P* < 0.05) negatively correlated with the genes involved in virulence (Table 3). As a result, the degraded soils were found with lower pH value and weakened activities of urease, sucrase and catalase, but with more abundant genes involved in stress responses and virulence when compared to the healthy soils.

## Discussion

In general, the long-term continuous monocropping with crops such as maize, sorghum, tobacco, *etc*., can cause severe soil degradation^17^. In this study, the field with long-term monocropping tobacco more than ten years was used as a model for studying the shifts of microbial composition and functional gene structure of degraded soils via metagenomics technology.

Firstly, we hypothesized the taxonomic, functional composition and structure of soil microbial communities would be shifted in the degraded soils. As expected, it was found the taxonomic composition of soil microbial communities dramatically shifted in the degraded soils. The abundance of *Blastococcus, Agromyces, Thermoleophilum, Gemmatimonas, Trichoderma* and *Aspergillus,* significantly increased in the healthy soils when compared to the degraded soils. *Blastococcus, Agromyces* and *Thermoleophilum* belong to *Actinobacteria*, and some species of *Agromyces* are found with nitrogen-fixing capacity^18^. Many *Actinobacteria* can produce antibiotics to inhibit plant pathogens in soil. *Gemmatimonas* contains purple bacterial photosynthetic reaction centers, which can participate in carbon cycling in soil^19^. *Trichoderma* spp. can produce antibiotics, trigger systemic resistance, improve plant nutrient uptake, parasitize other pathogenic fungi and compete with deleterious microorganisms in soil^20^. For example, *T. harzianum* produces phosphatase to scavenge, mobilize and acquisite phosphate for enhancing soil fertility and promoting plant growth^21^. *Aspergillus persii* is reported with a strong antibacterial activity to several plant pathogens such as *Xanthomonas arboricola*^22^. These beneficial microorganisms may improve the soil quality. However, the poor soil environments (acidification, poor nutrients, hardening, *etc*.) lead to a decrease of beneficial microorganisms and poor soil microbial community structure in the degraded soils (Fig. 1). Also, the decrease of beneficial microorganisms further aggravates the poor bio-functions of degraded soils (Table 1).

The abundance of pathogens such as *Ralstonia, Pseudomonas, Verticili, Rhizoctonia, Sclerotina* and *Fusarium* were higher in the degraded soils than the healthy soils. *Ralstonia solanacearum* is a pathogenic agent of bacterial wilt^23^; *Pseudomonas syringae, P. aeruginosa, P. corrugate* and *P. mediterranea* are economically important plant pathogens^24^,^25^,^26^; *Rhizoctonia solani* is a soil-borne fungal pathogen that causes disease in a wide range of plants including tobacco^27^; *Fusarium* contains many plant pathogens such as *F. oxysporumi* for infection of tomato and *F. solani* for infection of pea^28^. It is possible that these pathogenic microorganisms may cause serious plant diseases in the degraded soils, consistent with the high disease index of tobacco bacteria wilt in the degraded soils (Table 2).

GeoChip is a high-throughput powerful tool for studying the functional diversity and metabolic potential of microbial communities in a variety of environments^12^,^15^. Here, we analyzed the soil microbial functional genes using GeoChip 5.0. As a new generation of functional gene array, Geochip 5.0 contains 57,000 oligonucleotide probes and covers over 144,000 genes from 393 gene families, both are much more than the last generations of GeoChip^29^. We hypothesized the key genes related to stress responses, metal resistance, S cycle, virulence, and degradation of plant cell wall would be increased in the degraded soils. As expected, this hypothesis was verified by the increased functional metabolic potential of these genes in the degraded soils. Moreover, the increased functional metabolic potential of these genes tended to negatively influence on soil function and plant growth in the degraded soils.

The metabolic potentials of genes involved in metal resistance and stress response will be increased under acidic and metal-rich conditions for adaptation of stress from these extraordinary communities^30^. Likewise, we also found the functional metabolic potential of stress response genes were significantly higher in the degraded soils than the healthy soils. The stress response genes were stimulated and enhanced for adaption of adverse environmental conditions such as soil acidification in the degraded soils.

Soil acidification is found in many areas of cultivated land in China and renders land unsuitable for crops^31^,^32^,^33^. Soil pH seriously affects on plant growth, as many nutrients will become unavailable for plant at low pH. Here, we found that soil pH was lower in the degraded soils than the healthy soils. Available P in the degraded soils was significantly lower than the healthy soils (Table 1). Plant height and stem girth of tobacco grown in the healthy soil were significantly higher than the degraded soils (Table 2), indicating that the acidic degraded soils reduce the availability of plant nutrients and the growth of the plant. Also, the urease, sucrase and catalase activities were all lower in the degraded soils than in the healthy soils, indicating the soil biological activity is obviously decreased in the acidified and degraded soils. GeoChip analysis also showed the functional metabolic potential of genes involved in sulfur oxidation was higher in the degraded soils when compared to the healthy soils. Sulfur can be oxidized to sulfuric acid by sulfur oxidizing bacteria, so the sulfur oxidation is detrimental if the quantity of produced acid exceeds the buffering capacity of environment^34^,^35^. Here, it is speculated the higher functional metabolic potential of sulfide oxidation genes (*e.g., fccab* and *soxV*) is related to the acidification of degraded soils. Furthermore, the functional metabolic potential of sulfate reduction gene (*dsrA*) to catalyze H_2_S production was also enhanced in the degraded soils. H_2_S is highly toxic by inhibiting cytochrome C oxidase activity, blocking energy production, and inhibiting other metal-dependent enzyme activities in plants^36^,^37^. Thereby, the increased functional metabolic potential of S cycle genes may intensify the acidification of soil, especially in the degraded soils.

The pathogens virulence relies on a large number of virulence factors, including plant cell-wall degrading enzymes secreted by Type II secretion system^38^, and effectors secreted by type III and VI secretion systems^39^,^40^. Degradation of plant cell wall is an important aspect of pathogenesis. Pathogenic fungi secrete numerous enzymes for degrading cell wall such as polygalacturonases, exo-β-1,3-glucanases, xylanases and cellulases during the early stages of infection^41^,^42^. For example, chitinase is important for *F. oxysporum* virulence by hydrolyzing β-1,4 linkages in chitin polymers^43^. Here, we found the functional metabolic potential of plant cell wall degradation genes and virulence genes were both increased in the degraded soils, which are favorable for pathogens to infect tobacco in the field.

We also hypothesized that the weak soil enzyme activities and severe soil acidification in the degraded soils were closely related to the specific functional genes of microorganisms, which were verified with significant correlations among soil pH, soil enzyme activities (urease, sucrase and catalase) and functional genes. The S cycle related genes were significantly negatively related to the activities of urease and sucrase, and the weak activities of urease and sucrase were also related to the high functional metabolic potential of S cycle genes in the degraded soils. S cycle related genes (*dsra, soxV*) were also negatively related to soil pH. Thereby, the enhanced S cycle related genes may further weaken soil enzyme activities by acidification of soil in the degraded soils. Besides, the stress and virulence related genes were negatively related to soil pH and soil enzyme activities. The increased functional metabolic potential of stress and virulence related genes are related to decreased soil enzyme activities and soil pH in the degraded soils, which are possible to be used as important indicators for assaying soil quality.

In this study, we found a shift of soil microbial community composition and structure in the degraded soils with low soil pH and weak soil enzyme activities. Also, the functional metabolic potential of genes involved in stress, virulence, mental and S cycle linked to soil acidification and other adverse environmental conditions, were higher in the degrade soils than in the healthy soils. The degraded soils are not favorable for beneficial microorganisms but for pathogens, because the former are generally saprophytic microorganisms with a reliance on soil nutrients whereas the later are parasitic microorganisms without a strict reliance on soil nutrients. As a result, the increased pathogenic virulence genes and degradation of plant cell wall genes are very potential for increasing severe tobacco bacterial wilt in the degraded soils.

## Methods

### Experimental plots, tobacco growth and soil samples collection

We conducted this study with tobacco planting soils at Enshi County of Hubei Province, China. At Enshi County, the continuous monoculture of tobacco has caused serious soil degradation and soil acidification in the field. Total 10 plots containing five plots of healthy soils and five plots of degraded soils, were selected with a continuous cultivation of tobacco more than 10 years. The plots are yellow-brown soil in a subtropical humid climate (an annual rainfall of 1400–1500 mm and annual average temperature of 16 °C).

In the five plots of healthy soils, the tobacco plants grew well without pathogens infection. Conversely in the five plots of degraded soils, the soils were acidic, and the tobacco plants grew poorly with serious bacterial wilt caused by *R. solanacearum*. Soil samples (0–20 cm) were collected before transplantation. At each plot, soils were collected from 20 different sites and then mixed together before further analysis. Each sample was detected for the soil moisture content. After that, each soil sample was partitioned into two subsamples, with one subsample for soil DNA extraction and another for analysis of soil physical and chemical properties after being dried in open air.

At 120 d after transplantation, the height and stem circumference of tobacco were recorded in the selected 10 plots. On the other hand, total 60 tobacco seedlings were randomly selected from each plot to investigate the disease incidence and disease index of tobacco bacterial wilt as previously described^44^.

### Analysis of soil physical and chemical properties

After being suspended with water (soil:water = 1:2.5, w/v), the soil pH was measured using a pH meter. Soil organic matter was assayed using dichromate wet combustion^45^. Soil available nitrogen (AN) content, available phosphorus (AP) content, and available potassium (AK) content were analyzed as previously described^45^. The activity of urease, acid phosphatase, catalase and sucrase was detected according to Kandeler^46^, Tabatabai^47^ and Guan^48^, respectively. All tests were performed in triplicates.

### Soil DNA extraction

Soil microbial community DNA was extracted from soil samples using soil genome extraction Kit (FastDNA Spin Kit for soil, MP Biomedicals, USA). 0.4 g soil mixture was added with 978 μl sodium phosphate buffer following with vortex for 15 s, then added with 122 μl MT buffer supported by the Kit and shaken vigorously. Thereafter, the soil samples were homogenized in the FastPrep instrument for 40 s at a speed setting of 6.0, then the supernatant was loaded to the SPIN^TM^ filter tube and the soil DNA was eluted by 100 μl DES buffer supported by the Kit according to the instructions of manual. The extracted soil DNA quality was determined using a NanoDrop ND-1000 spectrophotometer (NanoDrop Technologies Inc., Wilmington, DE, USA), and the DNA concentration was quantified by PicoGreen using a FLUO star Optima instrument (BMG Labtech, Jena, Germany).

### MiSeq sequencing of 16S rRNA and 18S rRNA gene amplicons

16S rRNA gene amplicons for bacteria and 18S rRNA gene amplicons for fungi were sequenced at the Shanghai Biotechnology Corporation (Shanghai, China) by an Illumina MiSeq sequencing system. The extracted soil DNA was used as template to prepare 16S rRNA and 18S rRNA gene libraries. The V3 and V4 region of 16S rRNA genes were amplified with the primer pair 338F (5′-ACTCCTACGGGAGGCAGCA-3′) and 806R (5′-GGACTACHVGGGTWTCTAAT-3′). The V5-V7 region of 18S rRNA genes were amplified with the primer pair 0817F (5′-TTAGCATGGAATAATRRAATAGGA-3′) and 1196R (5′-TCTGGACCTGGTGAGTTTCC-3′). Sample libraries were generated from the purified PCR products. MiSeq Reagent Kit was used for 2 × 250 bp paired-ends sequencing on MiSeq machine (Illumina, San Diego, CA, USA). The operational taxonomic units (OTUs) were classified using mothur (http://www.mothur.org/) at the 97% similarity level^49^. In the pipeline of Mothur and QIIME, we resample same number of sequence per sample based on the minimum number of sequence in the dataset for analysis of OTU richness (Chao 1) and phylogenetic diversity (Shannon’s index). Sequence identities of rRNA gene fragments were analyzed by BLAST (NCBI).

### GeoChip analysis

GeoChip 5.0 was used to analyze soil microbial functional genes. GeoChip 5.0 was performed by Institute for Environmental Genomics and Department of Botany and Microbiology, University of Oklahoma (Norman, OK 73019, USA). The GeoChip 5.0 (60K arrays) used in this study contains more than 57,000 oligonucleotide probes, covering over 144,000 gene sequences from 393 gene families involved in the key biogeochemical cycles including carbon, nitrogen, and sulfur cycling, phosphorus utilization, metal resistance, antibiotic resistance, organic remediation, and other processes in various ecosystems. The purified soil DNA (500 ng) was labeled and then hybridized with GeoChip 5.0 at 67 °C and 20 rpm for 24 h in a hybridization oven (Agilent Technologies, Cornelius, OR, USA). After hybridization, the arrays were washed using the Agilent hybridization buffer at room temperature, scanned with a NimbleGen MS200 Microarray Scanner (Roche NimbleGen, Inc., Madison, WI, USA) and then analyzed as previously described^13^,^50^.

### Statistical analysis

Statistical differences among soil samples were analyzed by a one-way analysis of variance (ANOVA) and least-significant-difference (LSD) tests. Canonical correspondence analysis (CCA) and Mantel test were used to link variation within functional community to environmental variables. Detrended correspondence analysis (DCA) was used to analyze the changes of soil microbial community structure.

## Additional Information

**How to cite this article**: Zhang, H. *et al*. Microbial taxa and functional genes shift in degraded soil with bacterial wilt. *Sci. Rep.* **7**, 39911; doi: 10.1038/srep39911 (2017).

**Publisher's note:** Springer Nature remains neutral with regard to jurisdictional claims in published maps and institutional affiliations.

## Supplementary Material

Supplementary Information

## Figures and Tables

**Table 1:** Soil biological, chemical and physical properties.

Soil properties		Healthy soils	Degraded soils
Enzyme activities	Urease (mg NH_4_-N h^−1^ g^−1^soil)	0.36 ± 0.03 A	0.26 ± 0.03 B
	Phosphatase (μg p-nitrophenol h^−1^)	1.68 ± 0.18 a	1.71 ± 0.20 a
	Sucrase (mg/g)	47.84 ± 10.08 A	27.84 ± 5.74 B
	Catalase (mL/g)	2.92 ± 0.44 a	1.79 ± 0.75 b
pH and nutrients	pH	6.61 ± 0.15 a	5.98 ± 0.42 b
	Available N (mg/kg)	140.98 ± 13.23 a	139.72 ± 17.78 a
	Available P (mg/kg)	32.86 ± 3.63 a	23.60 ± 8.02 b
	Available K (mg/kg)	302.75 ± 92.47 a	306.33 ± 45.64 a
	Organic matter (g/kg)	30.22 ± 2.69 a	29.83 ± 3.33 a
physical properties	Soil bulk density (g/cm^3^)	1.15 ± 0.05 a	1.21 ± 0.07 a
	Soil moisture content (%)	21.59 ± 1.54 a	20.94 ± 1.07 a
	Total soil porosity (%)	55.27 ± 1.60 a	54.59 ± 2.55 a

Mean ± standard deviation (*n* = 5). Values within the same row followed by different lower-case letters differ at *P* < 0.05; Values within the same row followed by different upper-case letters differ at *P* < 0.01.

**Table 2:** Tobacco growth and disease incidence.

Treatments	Incidence of bacterial wilt (%)	Disease index	Plant height (cm)	Stem girth (cm)
Tobacco in degraded soils	85.48 ± 35.57 A	79.92 ± 34.26 A	97.23 ± 6.42 a	9.12 ± 0.44 a
Tobacco in healthy soils	0 ± 0 B	0 ± 0 B	110.65 ± 8.36 b	9.8 ± 0.23 b

Values are means (n = 5) ± standard error. Different lower-case letters within columns indicate significant differences (*P* < 0.05), and different upper-case letters within columns indicate very significant differences (*P* < 0.01).

**Table 3:** Correlations between microbial community functional genes and soil properties from Mantel test.

Gene	Urease		Sucrase		Catalase		pH	
	Statistic r	P	Statistic r	P	Statistic r	P	Statistic r	P
S cycle								
*cysJ*	−0.673	**0.033**	−0.555	0.096	−0.518	0.125	−0.611	0.061
*cysI*	−0.636	**0.048**	−0.467	0.173	−0.306	0.39	−0.516	0.127
*dsra*	−0.723	**0.018**	−0.643	**0.045**	−0.552	0.098	−0.639	**0.047**
*fccab*	−0.664	**0.036**	−0.635	**0.049**	−0.517	0.126	−0.594	0.07
*soxV*	−0.821	**0.004**	−0.740	**0.014**	−0.839	**0.002**	−0.729	**0.017**
Stress								
*nsrR*	−0.770	**0.009**	−0.694	**0.026**	−0.636	**0.048**	−0.659	**0.038**
*cydA*	−0.765	0.01	−0.719	**0.019**	−0.561	0.092	−0.701	**0.024**
*resE*	−0.605	0.064	−0.565	0.089	−0.727	**0.017**	−0.715	**0.02**
*narI*	−0.688	**0.028**	−0.599	0.067	−0.564	0.09	−0.646	**0.044**
*fnr*	−0.654	**0.04**	−0.576	0.081	−0.532	0.114	−0.646	**0.044**
*baeR*	−0.750	**0.012**	−0.666	**0.036**	−0.623	0.054	−0.674	**0.033**
*cpxA*	−0.729	**0.017**	−0.666		−0.603	0.065	−0.643	**0.045**
*pspC*	−0.742	**0.014**	−0.67	**0.034**	−0.609	0.062	−0.679	**0.031**
*proX*	−0.719	**0.019**	−0.66	**0.038**	−0.649	**0.042**	−0.687	**0.028**
*pstS*	−0.763	**0.01**	−0.679	**0.031**	−0.632	**0.05**	−0.664	**0.036**
*pspF*	−0.758	**0.011**	−0.659	**0.038**	−0.576	0.081	−0.639	**0.047**
*pstB*	−0.694	**0.026**	−0.55	0.099	−0.567	0.087	−0.702	**0.024**
*sodA*	−0.792	**0.006**	−0.746	**0.013**	−0.640	**0.046**	−0.686	**0.028**
*degP*	−0.572	0.084	−0.407	0.243	−0.572	0.084	−0.675	**0.032**
Virulence								
*fyuA*	−0.811	**0.004**	−0.683	**0.029**	−0.857	**0.002**	−0.856	**0.002**
*hopAF1*	−0.746	**0.013**	−0.576	0.082	−0.792	**0.006**	−0.715	**0.02**
*inv*	−0.735	**0.015**	−0.689	**0.027**	−0.621	0.056	−0.529	0.1
*iutA*	−0.751	**0.012**	−0.695	**0.026**	−0.918	**0**	−0.791	**0.006**
*las*	−0.843	**0.002**	−0.773	**0.009**	−0.799	**0.006**	−0.764	**0.01**
*npgA*	−0.774	**0.009**	−0.745	**0.013**	−0.684	**0.029**	−0.542	0.106

Bold texts indicate significant P-values (<0.05).
