# Supplementary material for: Microbial taxa and functional genes shift in degraded soil with bacterial wilt
Source: Sci Rep. 2017 Jan 4;7:39911. doi: 10.1038/srep39911 (PMC5209727; doi:10.1038/srep39911)

# Microbial taxa and functional genes shift in degraded soil with bacterial wilt

**Running title: Microbial communities respond to soil degradation**

Hongchun Zhang[1a], Rui Wang[1a], Shu Chen[1], Gaofu Qi[1], Zhili He[1,2], Xiuyun Zhao[1*]

[1]College of Life Science and Technology, Huazhong Agricultural University, Wuhan 430070, China;

[2]Institute for Environmental Genomics and Department of Microbiology and Plant Biology, University of Oklahoma, Norman, OK 73019, USA.

[a] Same contribution to this study.

*Correspondence author, E-mail: xiuyunzh@mail.hzau.edu.cn; Tel: +86-15387157410.

Table S1 Summary for 16S rRNA pyrosequencing data

| Samples | Number of reads | Number of OTUs | Chao 1 | Shannon’s index |
|---|---|---|---|---|
| D01 | 28837 | 6379 | 14135 | 7.54 |
| D02 | 25414 | 5948 | 14530 | 7.29 |
| D03 | 25119 | 5004 | 10076 | 7.15 |
| D04 | 28034 | 6364 | 12940 | 7.71 |
| D05 | 28034 | 6881 | 14886 | 7.76 |
| H01 | 44458 | 10566 | 53235 | 8.20 |
| H02 | 42545 | 7709 | 18288 | 7.96 |
| H03 | 38881 | 8233 | 22691 | 7.99 |
| H04 | 25645 | 9579 | 37568 | 8.13 |
| H05 | 35772 | 6888 | 14602 | 7.82 |

Table S2 Summary for 18S rRNA pyrosequencing data

| Sample | Number of reads | Number of OTUs | Chao 1 | Shannon’s index |
|---|---|---|---|---|
| H01 | 24900 | 1984 | 7336 | 4.29 |
| H02 | 43031 | 2131 | 9760 | 4.39 |
| H03 | 24976 | 1458 | 6643 | 3.65 |
| H04 | 25889 | 2216 | 10302 | 4.34 |
| H05 | 43600 | 1867 | 8443 | 3.99 |
| D01 | 43685 | 1524 | 6140 | 3.66 |
| D02 | 45838 | 1136 | 6314 | 3.77 |
| D03 | 28505 | 1729 | 8027 | 3.79 |
| D04 | 46567 | 1348 | 6633 | 3.63 |
| D05 | 38220 | 1776 | 8224 | 3.87 |

Table S3 Pathogenic *Ralstonia* and *Pseudomonas* OTUs

| OTU designation | Nearest strain (% identity) [a] | Abundance in Healthy soils [b] | Abundance in Degraded soils [b] |
|---|---|---|---|
| 890 | *Ralstonia solanacearum* strain in4ss52 (99%) | 6.6 ± 1.52 B | 15.6 ± 2.66 A |
| 10283 | *Ralstonia solanacearum* strain in4ss52 (98%) | 0.2 ± 0.45 b | 1.6 ± 0.89 a |
| 12313 | *Ralstonia solanacearum* strain 08BF31TG (99%) | 0.4 ± 0.54 B | 2.2 ± 0.84 A |
| 1075 | *Pseudomonas corrugate* strain BS3649 (99%) | 2.4 ± 0.54 B | 4.4 ± 0.89 A |
| 1197 | *Pseudomonas mediterranea* strain DSM 16733 (99%) | 2.2 ± 1.09 b | 4.2 ± 1.64 a |
| 4678 | *Pseudomonas aeruginosa* strain BOAKS 44E (99%) | 0.8 ± 0.44 b | 2.4 ± 1.51 a |
| 11417 | *Pseudomonas corrugata* strain BS3649 (99%) | 0.2 ± 0.44 B | 1.8 ± 0.83 A |
| 13587 | *Pseudomonas corrugata* strain BS3649 (98%) | 0.2 ± 0.44 B | 1.2 ± 0.44 A |
| 14466 | *Pseudomonas corrugata* strain BS3649 (98%) | 0 B | 1.4 ± 0.54 A |
| 23851 | *Pseudomonas syringae* isolate 21 (97%) | 0 B | 1.8 ± 0.83 A |

[a] % identity values are from analyses using BLAST (NCBI) where coverage was at least 97%.

[b] The different lower-case letters and capital letters in same row indicated significant difference between healthy and degraded soils at $P < 0.05$ and $P < 0.01$, respectively.

**Figure S1 Relative abundances of the dominant bacterial taxa in healthy and degraded soil samples at the phylum level.** H1-5: healthy soils; D1-5: degraded soils.

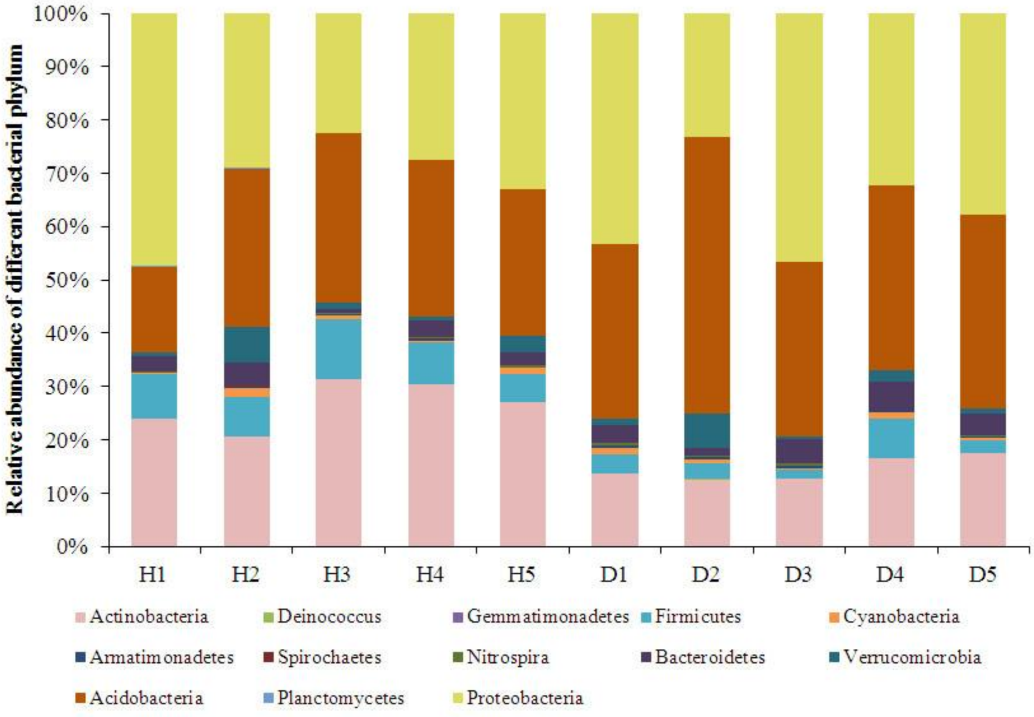


**Figure S2 Relative abundances of the dominant fungal taxa in healthy and degraded soil samples at the phylum level.** H1-5: healthy soils; D1-5: degraded soils.

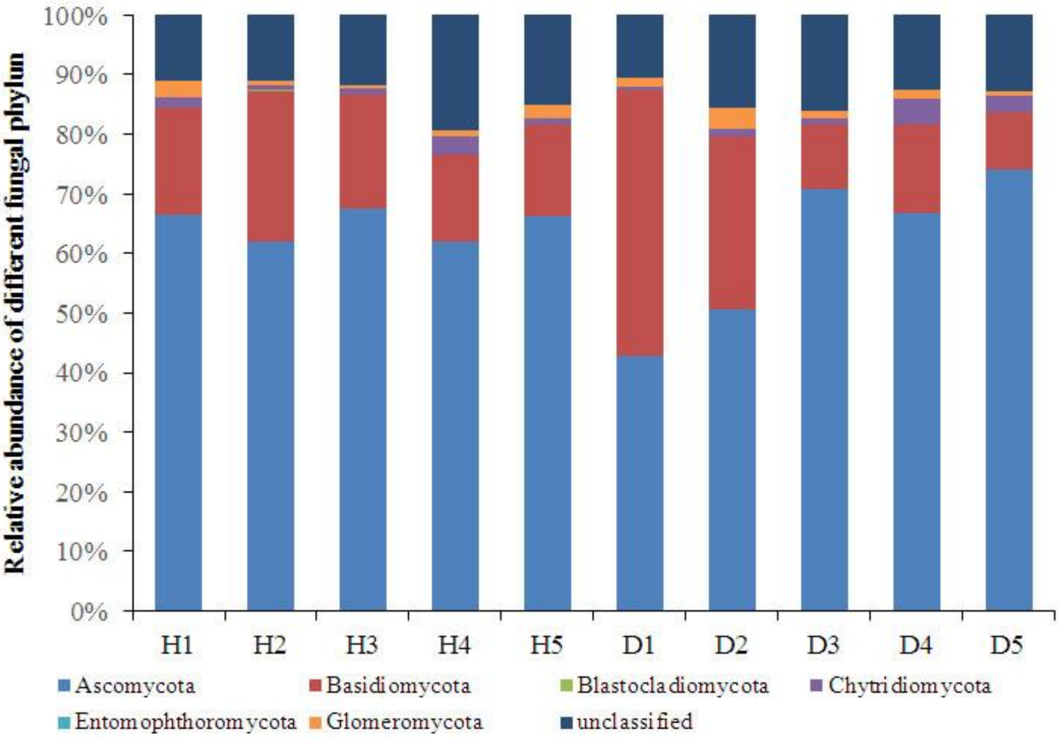

**Figure S3 Relative abundance of various functional groups detected in healthy and degraded soil samples.** H1-5: healthy soils; D1-5: degraded soils.

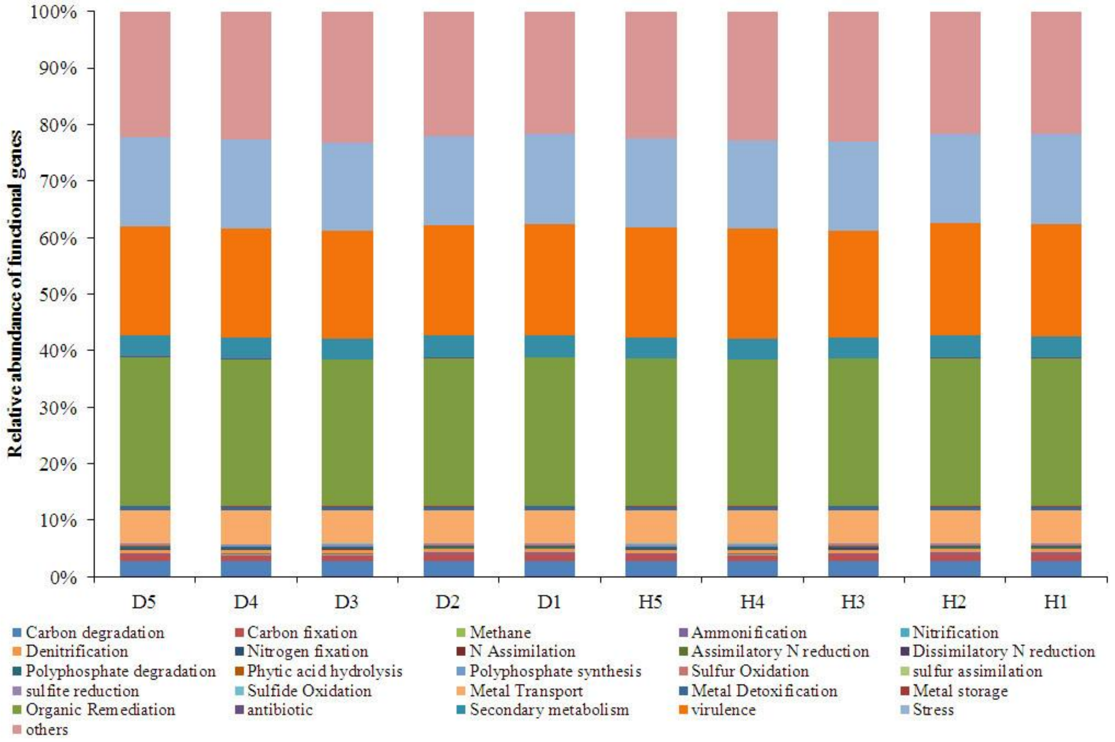


**Figure S4 Stress related genes.** The normalized signal intensity of functional genes involved in stress was compared between healthy and degraded soils. All data are presented as the mean ± SE. * $P < 0.05$.

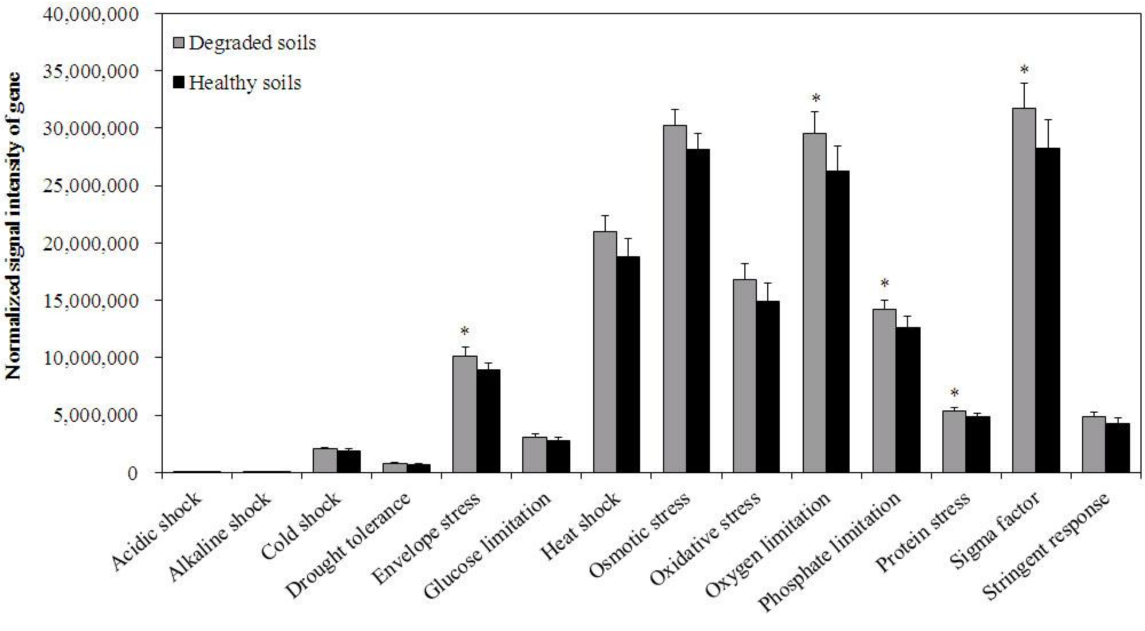

**Figure S5 Virulence related genes.** The normalized signal intensity of genes involved in virulence was compared between healthy and degraded soils. All data are presented as the mean ± SE. ** $P < 0.01$, * $P < 0.05$.

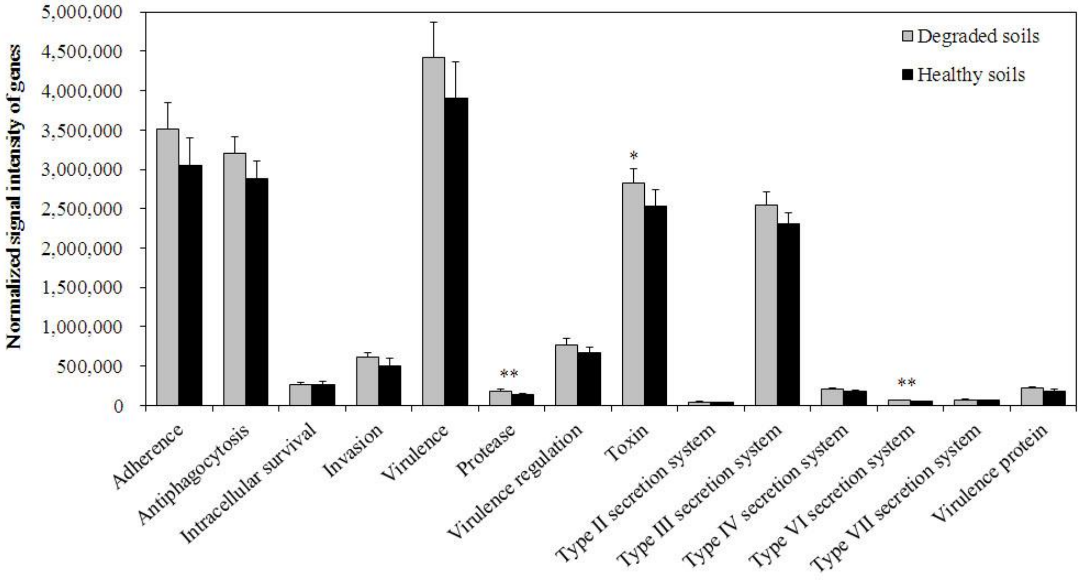


**Figure S6 Metal related genes.** The normalized signal intensity of metal resistance genes was compared between healthy and degraded soils. All data are presented as the mean ± SE. ** $P < 0.01$, * $P < 0.05$.

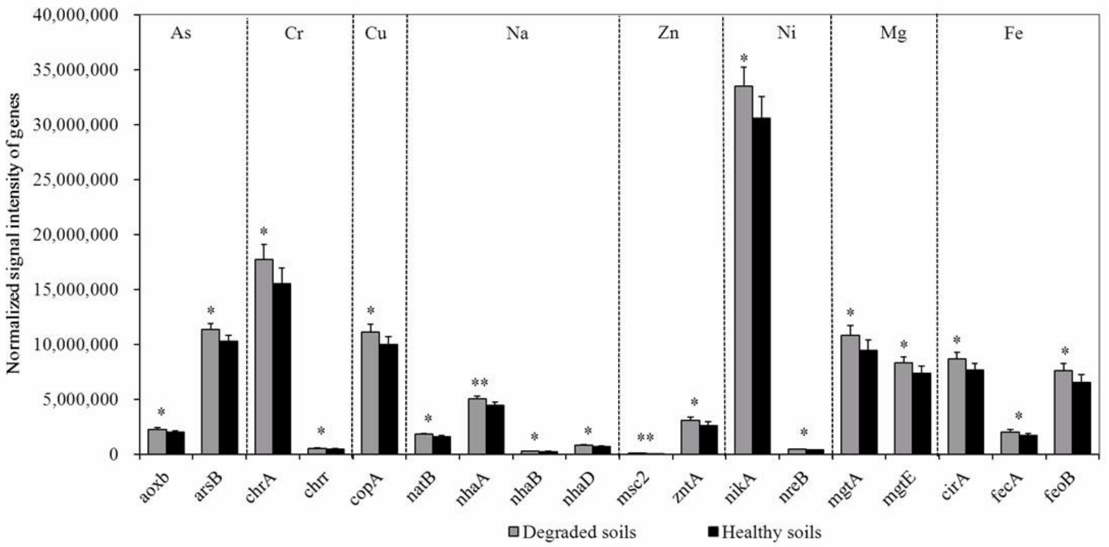

Supplement: Supplementary Information [file srep39911-s1.pdf]
